# Characteristics of interbody bone graft fusion after transforaminal lumbar interbody fusion according to intervertebral space division

**DOI:** 10.3389/fsurg.2022.1004230

**Published:** 2022-10-25

**Authors:** Songjie Xu, Lei Zang, Qian Lu, Peng Zhao, Qichao Wu, Xueming Chen

**Affiliations:** ^1^ Department of Orthopedics, Beijing Luhe Hospital, Capital Medical University, Bejing, China; ^2^ Department of Orthopedics, Beijing Chaoyang Hospital, Capital Medical University, Beijing, China

**Keywords:** lumbar vertebra, interbody fusion, CT, intervertebral space division, inner cage

## Abstract

**Background:**

According to intervertebral space division, the characteristics of interbody bone graft fusion after transforaminal lumbar interbody fusion (TLIF) were assessed *via* computed tomography (CT) scan to provide a theoretical basis for selecting the bone grafting site of interbody fusion.

**Methods:**

The medical records of 57 patients with lumbar spinal stenosis and disc herniation treated with TLIF were analysed retrospectively. In total, 57 segments received lumbar interbody fusion. A thin-layer CT scan was performed to evaluate fusion in each zone of the fusion space.

**Results:**

The fusion rates were 57.89% (*n* = 33) in the anterior cage zone, 73.68% (*n* = 42) in the posterior cage zone, 66.67% (*n* = 38) in the decompression zone, 26.32% (*n* = 15) in the contralateral decompression zone and 94.74% (*n* = 54) in the inner cage zone. There were significant differences among the fusion rates of the five zones (*P* < 0.001). Further pairwise comparison revealed that the fusion rates in the inner cage significantly differed from the anterior and posterior cages and decompression and contralateral decompression zones (*P* = 0.001, 0.002, 0.001 and 0.001, respectively).

**Conclusion:**

We think the central cage zone (i.e., inner cage) should be the focus of bone grafting. Although there is small volume of bone graft on the posterior cage zone, the fusion rate is relatively high, only secondary to the inner cage zone. The fusion rate is of the contralateral decompression zone is lower although there is a bone graft.

## Introduction

Lumbar interbody fusion (LIF) has developed into the standard of care for symptomatic lumbar spinal stenosis, spinal instability, spondylolisthesis, and degenerative scoliosis (1, 2). An interbody cage is commonly used during intraoperative fusion since it is important in achieving a stable interbody fusion of spinal units, restoring the lumbar lordosis and achieving a high interbody fusion rate in lumbar fusion surgery (3–5). However, surgical complications, including post-LIF non-union or pseudarthrosis, implant loosening, and cage subsidence, resulting in poor clinical outcomes, still remain a major challenge (6). Despite the decades of effort, most recent studies indicated that the non-union rates after LIF still ranged from 7% to 20%, with a significantly higher incidence in cases spanning 3 or more spinal levels (7–10).

In order to improve the fusion rate, some scholars began to pay attention to the location of fusion after LIF. Seo (11) found that the fusion rate for the inner cage area reached 100% after posterior lumbar interbody fusion (PLIF). However, the fusion in the lateral space outside of cages was not satisfactory, though reasonable (72.3%). Transforaminal lumbar interbody fusion (TLIF) approach has satisfactory clinical outcomes and offers various potential benefits over conventional posterior lumbar interbody fusion, including an increased fusion surface area, less blood loss, less wound infections, less reoperation, less retraction on the thecal sac and conus medullaris, lower incidence of neural element injury and lower subsidence (11–14). Ghasemi et al. reported that TLIF was superior to PLF with respect to functional outcome and fusion rate (92% vs. 81%) (15). Plantz also found no significant difference in PRO at two-year follow-up between PLIF and TLIF for the treatment of lumbar disc degeneration. PLIF is associated with a five times higher risk of dural tears (16). Chi (2) also believed that PLIF should be avoided in the management of lumbar degenerative disc disease due to the inferiority of overall complications, and TLIF seems to have the safest profile in terms of neural, spinal, and vascular events.

With minimally invasive fusion technology, the operator can achieve fusion with less trauma and bone grafting. However, there have been few studies about the specific location of fusion after TLIF. Hence, this study aimed to identify the fused segments in each zone on the intervertebral space plane after open TLIF, analyse and compare the fusion rates of different zones and explore the zone with a high fusion rate.

## Materials and methods

### Patients

57 patients with lumbar degenerative diseases who underwent posterior lumbar spinal canal decompression, transforaminal interbody fusion, bone grafting and internal fixation at our hospital from March 2013 to August 2017 were retrospectively included. The inclusion criteria were patients with diagnosis of single-segment lumbar degenerative diseases, including lumbar spinal stenosis (LSS), lumbar disc herniation (LDH) and lumbar spondylolisthesis (grade I); and those who did not respond to non-surgical treatment for more than half a year and who experienced disease recurrence; and those who underwent single-segment TLIF. The exclusion criteria were patients with previous lumbar surgery history, severe lumbar deformity, lumbar spondylolisthesis and spondylolysis, osteoporosis or ankylosing spondylitis and other medical conditions that were not suitable for surgical treatment.

The fused segments were distributed as follows: L1–2, L2–3, L3–4, L4–5 and L5–S1 in 1, 5, 4, 36 and 11 patients, respectively. All patients provided written informed consent, and this study protocol was performed in accordance with the Declaration of Helsinki reviewed and was approved by the Ethics Committee of The Hospital.

### Surgical methods

The operations were performed by the same surgeon with more than 10 years of experience in lumbar interbody fusion. The surgery was performed with the patient in the prone position under general anesthesia (17). A posterior median approach was used to expose the vertebral plate and the articular process of the operative segment. Firstly, four pedicle screws were used. Then, the upper and lower vertebral plates and unilateral facet joints of the operative segment were removed to expose the dural sac and nerve root and protect the nerve. Afterwards, the intervertebral disc was taken out, and the cartilage plates of the upper and lower vertebral bodies were removed with a ring curette. Subsequently, the resected autologous bone was sheared and transplanted into the intervertebral space. Then, an appropriately sized cage (Johnson / Johnson) made of PEEK material was placed into the autologous bone, which was then set in the intervertebral space, with a total bone graft volume of 5–10 (mean: 7) ml. Finally, the fixing rod was installed, and the screws were tightened after longitudinal pressurisation between them.

### Division of fusion zones

**Figure 1:**
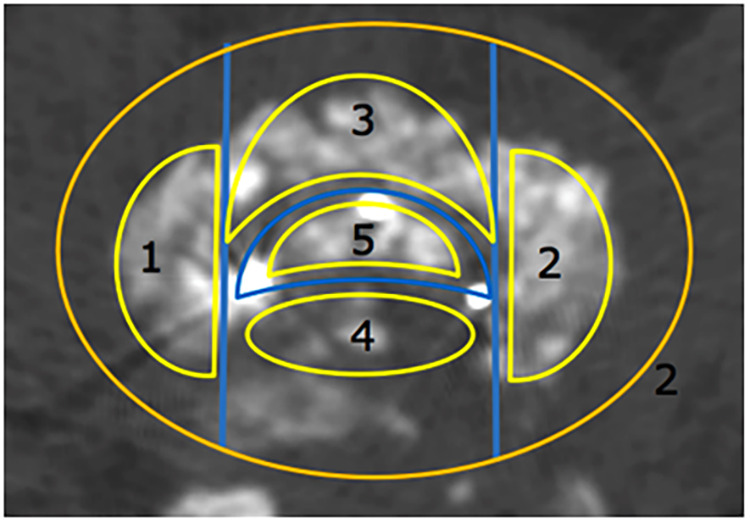
Fusion area in the implanted cage. 1: Decompression zone. 2: Contralateral decompression zone. 3: Anterior cage zone. 4: Posterior cage zone. 5: Inner cage zone.

The fusion area was divided into five zone based on the markers in the cage and the challenges in making bone beds during surgery. The bone graft area in the cage was marked as the inner cage zone, the anterior cage area (ventral) as the anterior cage zone, the posterior cage area (dorsal) as the posterior cage zones, the decompression side of the cage as the decompression zone and the contralateral decompression side as the contralateral decompression zone (Figure 1).

### Determination of intervertebral fusion

The fusion space and successful fusion were assessed *via* sagittal 2D lumbar CT scan reconstruction (18), and fusion was defined as the presence of trabecular bridging (19, 20).

### Evaluation method

**Figure 2:**
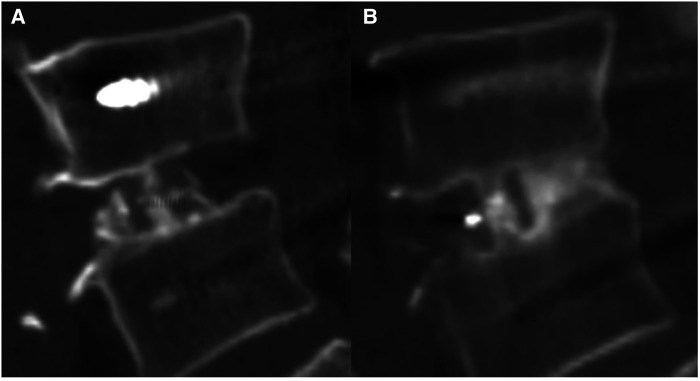
A 71-year-old Male patient received cage implantation after surgery for lumbar spinal stenosis. (**A**) Sagittal lumbar CT scan performed at postoperative 3 days revealed good bone filling in the inner cage zone but no bone graft in the posterior cage zone. (**B**) CT at postoperative 3 years showed bone remodelling and passage of the bone trabecula in the inner cage zone and the posterior cage zone.

**Figure 3:**
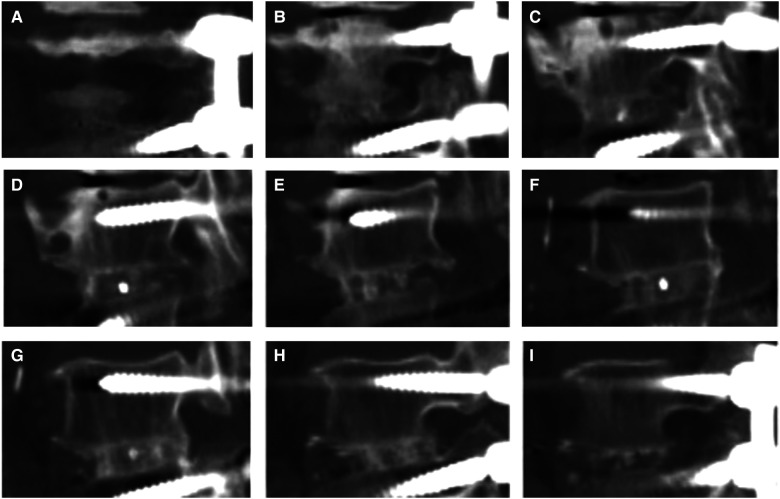
For lumbar 4-5 TLIF, the decompression side is on the left, and 2D CT reconstruction in our hospital was scanned from right to left. From (**A**–**C**), the opposite side of decompression was observed continuously from the first image until the appearance of the cage marker. (**D–F**) is the front, inside and back areas of the cage, and the marker was observe from one side of the cage to the other side. (**G–I**) showed the decompression area, and the last image was observed after the marker on the other side of cage disappeared.

Fusion, which was defined as the clear growth of the trabecular bone into the upper and lower vertebral bodies in any zone (21), was evaluated *via* a 1-mm thin-layer CT scan (Figure 2). The fusion in each zone was assessed *via* sagittal two-dimensional CT scan reconstruction (Figure 3). Non-fusion was defined as the absence of continuous growth of the trabecular bone into the upper and lower vertebral bodies at any level (13, 14). Three senior spine surgeons performed the evaluation, of whom two reached a consensus regarding the final grade of each case. CT scan was used to evaluate the fusion rate after TLIF, and patients with follow-up time for at least 1 year after TLIF were included to improve the accuracy and rationality of the evaluation.

### Clinical assessment

All patients underwent a three-dimensional CT scan and reconstruction of the lumbar spine during follow-up (mean, 2.5 years; range, 1–5.5 years). Clinical outcomes of patients in our study were assessed by visual analog scale (VAS), oswestry disability index (ODI) and Japanese Orthopaedic Association (JOA) scores. VAS of leg pain, ODI scores and JOA scores were recorded before surgery, 1-week post operation and at the last follow-up.

### Statistical analysis

Statistical analyses were performed using SPSS 24.0 software (IBM SPSS, the USA). Continuous numerical data were expressed as means ± SD and categorical data were expressed as percentages (%). The fusion rates of the five zones were compared using the chi-square test. The comparison between every two zones was corrected using the *Bonferroni* method, and then were analysed using the chi-square test. Repeated measures analysis of variance (ANOVA) was used to analyse VAS of leg pain, ODI scores and JOA scores before surgery, postoperative 1-week and at the last follow-up. Two-tailed probability value of *P* < 0.05 was considered as statistically significant.

## Results

The baseline data were shown in Table 1. In total, there were 25 men and 32 women aged 25–78 (mean: 57.9) years.

**Table 1:** Characteristics of patients.

Total patients (*n*)	*n* = 57
Male, *n* (%)	25 (43.86)
Age (years)	57.93 ± 12.71
BMI	25.0 ± 3.54
BMD	−0.6 ± 1.8
Follow-up time (years)	2.49 ± 1.29
Level, *n* (%)	
L1–2	1 (1.75)
L2–3	5 (8.77)
L3-4	4 (7.02)
L4–5	36 (63.16)
L5–S1	11 (19.30)
Diagnosis, *n* (%)	
Lumbar spinal stenosis	38 (66.67)
Lumbar disc herniation	6 (10.53)
Lumbar spondylolisthesis	13 (22.80)

BMI, Body mass index; BMD, Bone mineral density.

**Figure 4:**
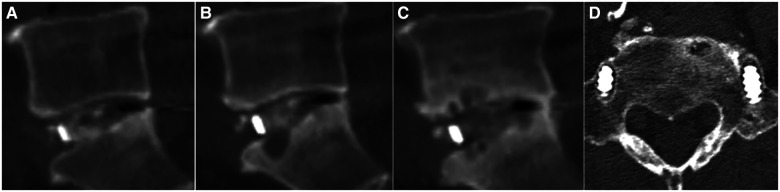
A 69-year-old Male patient with lumbar spinal stenosis underwent cage implantation. (**A**) Sagittal lumbar CT scan performed at postoperative 3 days showed good bone filling in the inner cage zone and no bone graft in the posterior cage zone. (**B**) CT scan performed at postoperative 3 months showed cage subsidence and educed upper and lower endplate space in the posterior zone. (**C**) CT scan performed at postoperative 2 years showed a lucent line without bone remodelling or passage of the bone trabecula in the inner cage zone. However, complete bone remodelling and passage of the bone trabecula in the posterior cage zone were observed. (**D**) Cross-sectional lumbar CT scan performed at postoperative 2 years showed loosening of the pedicle screws.

The fusion rates of the five zones were assessed by comparing the fusion of the same segment in each patient. The fragment was considered fused if any of the five zones met the fusion criteria. A total of 55 fused segments (98.2%) were observed. Only one patient who received cage implantation did not achieve fusion in any zone. Table 2 show the cage fusion rates of the five zones measured *via* CT scan. The inner cage zone (*n* = 54, 94.74%) had the highest fusion rate, followed by the posterior cage zone (*n* = 42, 73.68%) and the contralateral decompression zone (*n* = 15, 26.32%). The fusion rates of the five groups were significantly different (*P* < 0.001). Non-fusion in the cage was observed in three patients. Among them, one presented with non-fusion in the five zones accompanied by bilateral pedicle screw loosening at L5. The other two patients achieved fusion in the posterior cage but not in the cage, and one experienced pedicle screw loosening (Figure 4).

**Table 2:** Comparison of the fusion rates in the five zones.

Patients, *n* = 57	Inner cage	Anterior cage	Posterior cage	Decompression	Contralateral	*P*-value
Patients, *n* (%)						*P *< 0.001
Fusion	54 (94.74)**	33 (57.89)*,**	42 (73.68)*,**	38 (66.67)*,**	15 (26.32)*	
Non-fusion	3 (5.26)	24 (42.11)	15 (26.32)	19 (33.33)	42 (73.68)	

* Indicate comparison with inner cage, *P* < 0.05.

** Indicate comparison with contralateral, *P* < 0.05.

Further pairwise comparison revealed that the fusion rates in the inner cage significantly differed from that in the anterior and posterior cages and decompression and contralateral decompression zones (*P *= 0.001, 0.002, 0.001 and 0.001, respectively). The fusion rate in the contralateral decompression zone significantly differed from that in the decompression and anterior and posterior cage zones (*P* = 0.001, 0.001 and 0.001, respectively) (Table 2).

The VAS of leg pain, ODI scores and JOA scores of patients at postoperative 1 week and the last follow-up were significantly lower than those before surgery (*P* < 0.05). The ODI scores and JOA scores of patients at the last follow-up were significantly improved than their 1-week postoperative scores (*P* < 0.05) (Table 3).

**Table 3:** VAS of leg pain, ODI score and JOA score.

	Preoperative	Postoperative 1 week	Last follow-up	*P*-value
VAS of leg pain	6.16 ± 1.65	1.81 ± 1.08*	1.37 ± 1.01*	*P *< 0.001
ODI score	48.79 ± 8.39	17.86 ± 4.57*	11.05 ± 3.87*,**	*P *< 0.001
JOA score	13.32 ± 4.09	22.15 ± 5.33*	27.30 ± 5.35*,**	*P *< 0.001

VAS, Visual analog scale; ODI, Oswestry disability index; JOA, Japanese Orthopaedic Association score.

* Indicate comparison with preoperative, *P *< 0.05.

** Indicate comparison with postoperative 1 week, *P *< 0.05.

## Discussion

Spinal fusion has been used for degenerative spinal diseases since Albea and Hibbs first applied it to spinal tuberculosis in 1911. Among different fusion technologies, TLIF is widely applied due to its high fusion rate (22). CT scan has been widely used in postoperative fusion evaluation because it can perform thin-layer scanning and multi-dimensional reconstruction, which is superior to static and dynamic plain film evaluation (23). Lee (24) found that after implantation of the cage filled with local bone chips, the fusion rate at postoperative 1 year was higher than that at postoperative 6 months, which maybe because the implanted local bone chips grew slower into the callus than the implanted iliac bone. Therefore, it is more accurate to measure the fusion rate at least postoperative 1 year. In our study, CT scan was used to evaluate the fusion rate after TLIF, and patients with follow-up time for at least 1 year after TLIF were included to improve the accuracy and rationality of the evaluation.

In this study, the fusion rate was 98.2%, which was consistent with previous reports (90%–100%) (25–28). Only one of 57 patients with LDH did not achieve fusion in every zone. This patient was re-visited at postoperative 1 year after surgery due to lumbar pain and discomfort, and a lumbar CT scan revealed bilateral pedicle screw loosening at L5, which might be correlated with osteoporosis and obesity. His condition improved after continuous use of a brace for protection. However, some studies reported low fusion rates. Giorgi's (29) reported an interbody fusion rate of 72.6% at postoperative 1 year in their prospective multicentre study, with 182 patients treated with TLIF were included. He believed that may be due to a postoperative follow up that was too short to determine definitive successful or unsuccessful fusion. He believes this may be due to the short postoperative follow-up time to determine whether fusion was successful.

In the past, some scholars used intervertebral space partition to study the characteristics of intervertebral fusion. Abbushi (30) divided intervertebral space into 16 areas in an average way of 4 × 4 to observe the spatial position of the fusion device after placement. Similarly, Choi (31) divided the intervertebral space into 9 areas in an average way of 3 × 3. However, they did not apply partition to evaluate the fusion situation, and such mechanical partition method could not accurately describe the fusion characteristics of each partition from CT. Lee (32) used two cages as a reference point to divide the intervertebral space into 7 regions to observe the characteristics of intervertebral fusion after TLIF, including between two cages, left side of left cage, right side of right cage, front of cage, back of cage and inner cage. However, since Lee's research objects were TLIF patients after implantation of two fusion devices, his results cannot represent the fusion characteristics after single-cage TLIF, which are more commonly used now.

The endplate preparation during TLIF has its unique characteristics. The operation blind area is prone to occur. The bone bed on the side of the fusion device is the easiest to clean, while the bone bed on the opposite side of the approach is the most difficult to handle, and cartilage residue is prone to occur, thus affecting the fusion effect (33). Therefore in this study, disc space is divided into five zones according to the difficulty of endplate preparation and metal marking point of cage: the inner cage zone, the anterior cage zone, the posterior cage zones, the decompression zone and the contralateral decompression zone. This partition is easier to identify when evaluating fusion with CT, which is simple, reproducible and helpful to guide the selection of key areas of bone grafting and clinical implementation.

In the five zones, the inner cage zone had the highest fusion rate (94.74%), which is consistent with the study of Seo (11), which divided the disc space implanted with the double cage undergoing PLIF into 4 zones and found that inner double cage zone had the highest fusion rates of 100%. This can be explained by Wolf's law (34). That is, appropriate stress is required during bone remodelling, and the cage and its internal bone graft play the main supporting role and bear more stress. Hence, callus formation is better. By contrast, the cage-like structure prevents the overflow of implanted bone chips, and high-density bone chips in a closed cage is conducive for enhancing fusion (35). In addition, during cage placement, the cage will cause friction with the endplate, which is equivalent to the secondary endplate preparation.

In our research, posterior cage zone is the second highest area, which is consistent with the study of Kim and Burkus (36). We think there are three reasons of high fusion rate in posterior cage zone. First is appropriate endplate preparation of the posterior endplate (12). To achieve spinal interbody fusion, a complete endplate preparation is essential to assure bone growth in the intervertebral space (21). The second reason is that there is more space in posterior cage zone for bone grafting than in other zones. Cages of TLIF are designed to be positioned along this anterior apophyseal ring or designed obliquely overlay the central portion of the disk, so there is plenty of space for a bone graft (35). The third reason is posterior cage zone is the most stable zone. This zone was supported by a cage in the anterior part and was fixed by a pedicle screw system in the posterior part, so this zone is more stable biomechanically. Furthermore, the stimulation of local hematoma enhanced the formation of active bone tissues. The living bone cells in the cages are exposed to the peri-implant hematoma and will eventually result in ossification around the cages (35). In our study, although there was no effective bone graft in the posterior cage zone, however, a clear fusion could be achieved in the posterior cage zone in many cases.

The contralateral decompression zone outside the cage had the lowest fusion rate, which was associated with insufficient preparation of the endplate and bone grafting. When nucleus pulposus is mixed with the autogenous bone graft, it can delay or decrease the bone formation inside the disc space, thus influencing the final fusion (37). Yao (38) found that bone grafting in the contralateral decompression zone was, indeed, poor in measuring the bone grafting area *via* CT scan after surgery. Some bone grafts achieve bone resorption at the final follow-up (35), particularly in the anterior decompression zone of the cage.

The current study had several limitations. Firstly, the sample size was small. Secondly, the follow-up period was short, and the study only focused on fusion during the last follow-up. Thirdly, the sequence of bony fusion in each zone was not described. Fourthly, the use of PEEK cages might decrease the fusion rate due to its chemically inert, as previous reported (22). However, this issue will be addressed in our subsequent study.

## Conclusions

This study first described the achievement of interbody fusion after TILF with single cage implantation. We think the central cage zone (i.e., inner cage) should be the focus of bone grafting. Bone fusion in the posterior cage zone even without bone grafting indicates that the endplate should be adequately prepared to achieve full fusion in the intervertebral space and to ensure the long-term efficacy of the surgery. Therefore, it may be more important to properly prepare the endplate in the bone grafting area and create a good environment for callus growth. There are no excessive surgical requirements for areas that are challenging to manage and those with bone grafts.

## Data Availability

The raw data supporting the conclusions of this article will be made available by the authors, without undue reservation.
